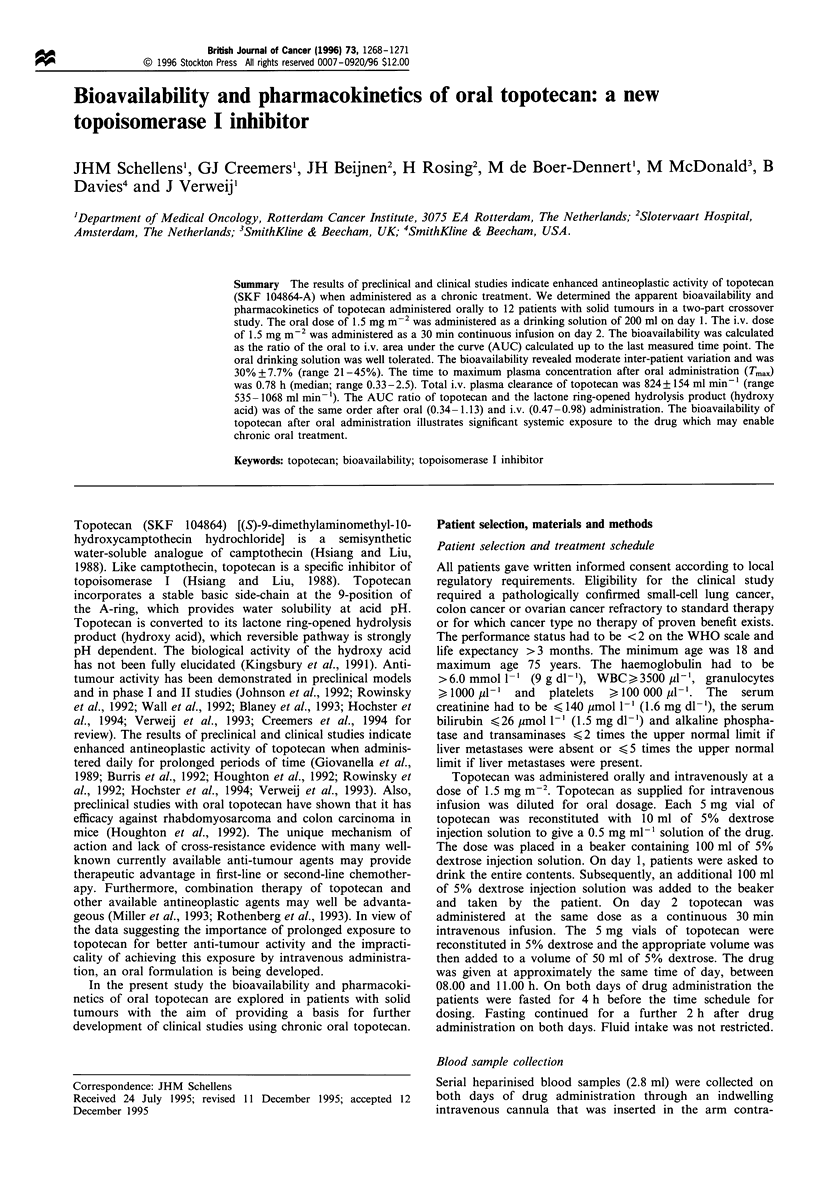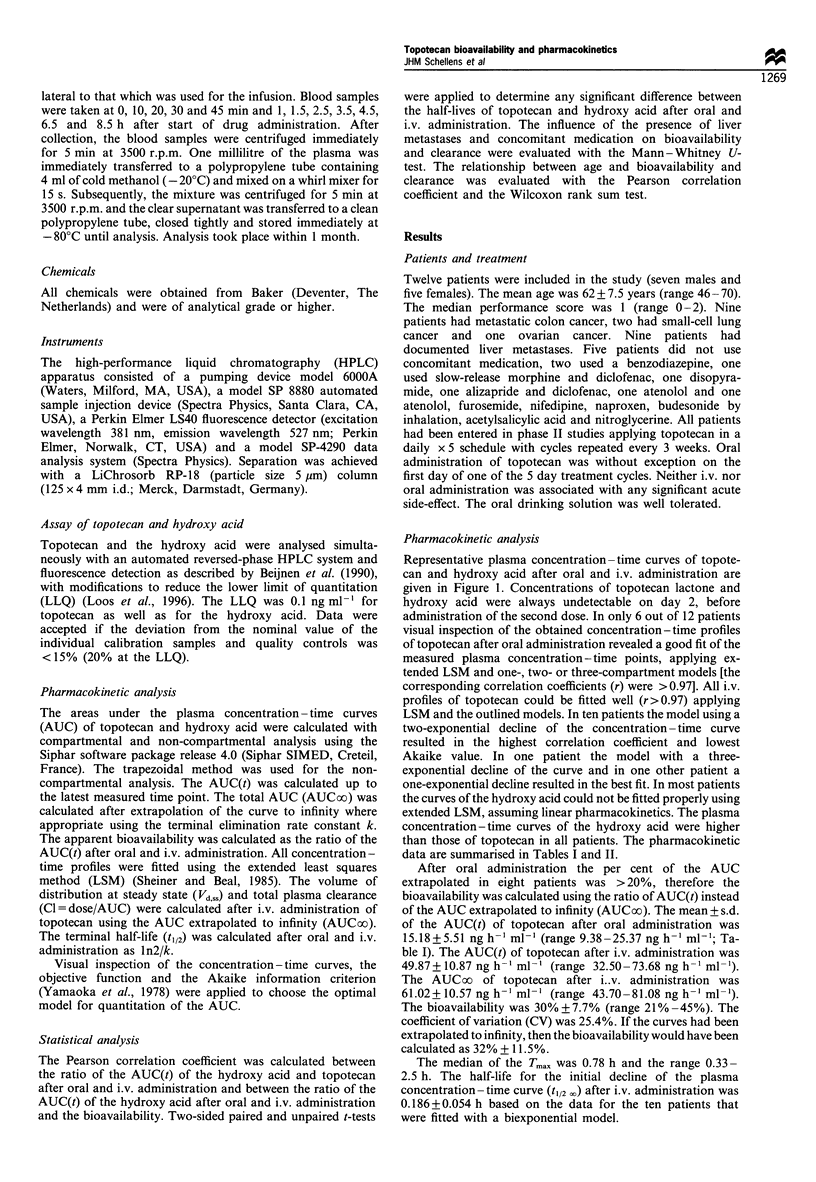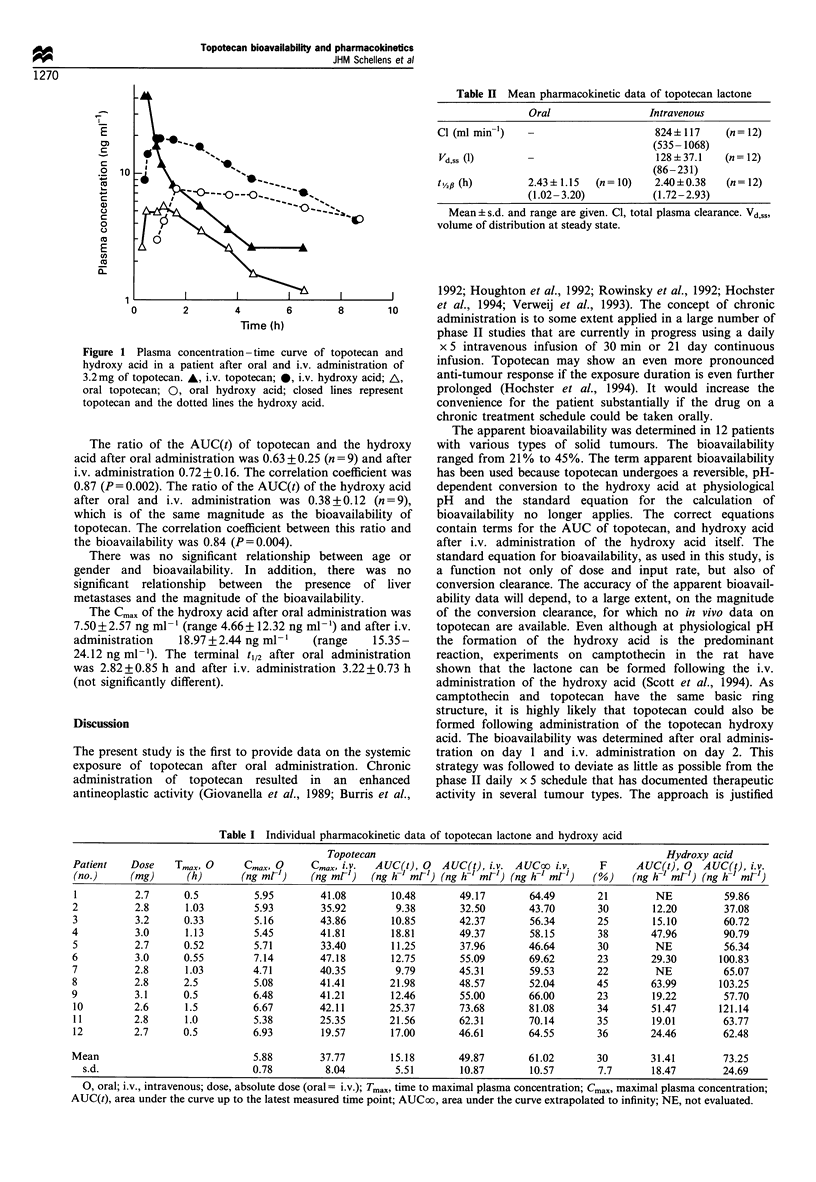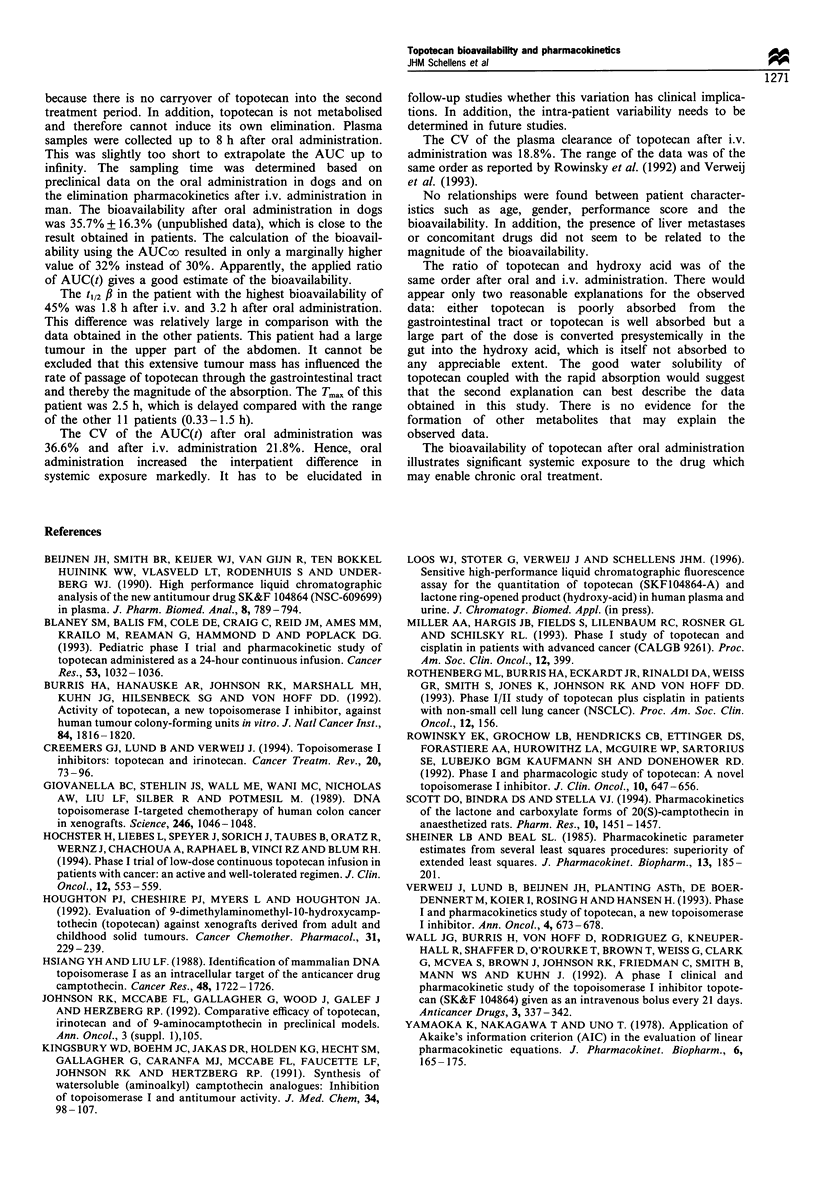# Bioavailability and pharmacokinetics of oral topotecan: a new topoisomerase I inhibitor.

**DOI:** 10.1038/bjc.1996.243

**Published:** 1996-05

**Authors:** J. H. Schellens, G. J. Creemers, J. H. Beijnen, H. Rosing, M. de Boer-Dennert, M. McDonald, B. Davies, J. Verweij

**Affiliations:** Department of Medical Oncology, Rotterdam Cancer Institute, The Netherlands.

## Abstract

The results of preclinical and clinical studies indicate enhanced antineoplastic activity of topotecan (SKF 104864-A) when administered as a chronic treatment. We determined the apparent bioavailability and pharmacokinetics of topotecan administered orally to 12 patients with solid tumours in a two-part crossover study. The oral dose of 1.5 mg m-2 was administered as a drinking solution of 200 ml on day 1. The i.v. dose of 1.5 mg m-2 was administered as a 30 min continuous infusion on day 2. The bioavailability was calculated as the ratio of the oral to i.v. area under the curve (AUC) calculated up to the last measured time point. The oral drinking solution was well tolerated. The bioavailability revealed moderate inter-patient variation and was 30% +/- 7.7% (range 21-45%). The time to maximum plasma concentration after oral administration (Tmax) was 0.78 h (median; range 0.33-2.5). Total i.v. plasma clearance of topotecan was 824 +/- 154 ml min-1 (range 535-1068 ml min(-1)). The AUC ratio of topotecan and the lactone ring-opened hydrolysis product (hydroxy acid) was of the same order after oral (0.34-1.13) and i.v. (0.47-0.98) administration. The bioavailability of topotecan after oral administration illustrates significant systemic exposure to the drug which may enable chronic oral treatment.


British Journal of Cancer (1996) 73, 1268-1271
© 1996 Stockton Press All rights reserved 0007-0920/96 $12.00

### Bioavailability and pharmacokinetics of oral topotecan: a new topoisomerase I inhibitor

JHM Schellens[1], GJ Creemers[1], JH Beijnen[2], H Rosing[2], M de Boer-Dennert[1], M McDonald[3], B Davies[4] and J Verweij[1]

*[1]Department of Medical Oncology, Rotterdam Cancer Institute, 3075 EA Rotterdam, The Netherlands; [2]Slotervaart Hospital, Amsterdam, The Netherlands; [3]SmithKline & Beecham, UK; [4]SmithKline & Beecham, USA.*

**Summary** The results of preclinical and clinical studies indicate enhanced antineoplastic activity of topotecan (SKF 104864-A) when administered as a chronic treatment. We determined the apparent bioavailability and pharmacokinetics of topotecan administered orally to 12 patients with solid tumours in a two-part crossover study. The oral dose of 1.5 mg $m^{-2}$ was administered as a drinking solution of 200 ml on day 1. The i.v. dose of 1.5 mg $m^{-2}$ was administered as a 30 min continuous infusion on day 2. The bioavailability was calculated as the ratio of the oral to i.v. area under the curve (AUC) calculated up to the last measured time point. The oral drinking solution was well tolerated. The bioavailability revealed moderate inter-patient variation and was 30% ± 7.7% (range 21–45%). The time to maximum plasma concentration after oral administration ($T_{max}$) was 0.78 h (median; range 0.33–2.5). Total i.v. plasma clearance of topotecan was 824 ± 154 ml $min^{-1}$ (range 535–1068 ml $min^{-1}$). The AUC ratio of topotecan and the lactone ring-opened hydrolysis product (hydroxy acid) was of the same order after oral (0.34–1.13) and i.v. (0.47–0.98) administration. The bioavailability of topotecan after oral administration illustrates significant systemic exposure to the drug which may enable chronic oral treatment.

**Keywords:** topotecan; bioavailability; topoisomerase I inhibitor

Topotecan (SKF 104864) [(*S*)-9-dimethylaminomethyl-10-hydroxycamptothecin hydrochloride] is a semisynthetic water-soluble analogue of camptothecin (Hsiang and Liu, 1988). Like camptothecin, topotecan is a specific inhibitor of topoisomerase I (Hsiang and Liu, 1988). Topotecan incorporates a stable basic side-chain at the 9-position of the A-ring, which provides water solubility at acid pH. Topotecan is converted to its lactone ring-opened hydrolysis product (hydroxy acid), which reversible pathway is strongly pH dependent. The biological activity of the hydroxy acid has not been fully elucidated (Kingsbury *et al*., 1991). Anti-tumour activity has been demonstrated in preclinical models and in phase I and II studies (Johnson *et al*., 1992; Rowinsky *et al*., 1992; Wall *et al*., 1992; Blaney *et al*., 1993; Hochster *et al*., 1994; Verweij *et al*., 1993; Creemers *et al*., 1994 for review). The results of preclinical and clinical studies indicate enhanced antineoplastic activity of topotecan when administered daily for prolonged periods of time (Giovanella *et al*., 1989; Burris *et al*., 1992; Houghton *et al*., 1992; Rowinsky *et al*., 1992; Hochster *et al*., 1994; Verweij *et al*., 1993). Also, preclinical studies with oral topotecan have shown that it has efficacy against rhabdomyosarcoma and colon carcinoma in mice (Houghton *et al*., 1992). The unique mechanism of action and lack of cross-resistance evidence with many well-known currently available anti-tumour agents may provide therapeutic advantage in first-line or second-line chemotherapy. Furthermore, combination therapy of topotecan and other available antineoplastic agents may well be advantageous (Miller *et al*., 1993; Rothenberg *et al*., 1993). In view of the data suggesting the importance of prolonged exposure to topotecan for better anti-tumour activity and the impracticality of achieving this exposure by intravenous administration, an oral formulation is being developed.

In the present study the bioavailability and pharmacokinetics of oral topotecan are explored in patients with solid tumours with the aim of providing a basis for further development of clinical studies using chronic oral topotecan.

Correspondence: JHM Schellens
Received 24 July 1995; revised 11 December 1995; accepted 12 December 1995

### Patient selection, materials and methods

### *Patient selection and treatment schedule*

All patients gave written informed consent according to local regulatory requirements. Eligibility for the clinical study required a pathologically confirmed small-cell lung cancer, colon cancer or ovarian cancer refractory to standard therapy or for which cancer type no therapy of proven benefit exists. The performance status had to be <2 on the WHO scale and life expectancy >3 months. The minimum age was 18 and maximum age 75 years. The haemoglobulin had to be >6.0 mmol $l^{-1}$ (9 g $dl^{-1}$), WBC ⩾ 3500 $\mu l^{-1}$, granulocytes ⩾ 1000 $\mu l^{-1}$ and platelets ⩾ 100 000 $\mu l^{-1}$. The serum creatinine had to be ⩽ 140 $\mu$mol $l^{-1}$ (1.6 mg $dl^{-1}$), the serum bilirubin ⩽ 26 $\mu$mol $l^{-1}$ (1.5 mg $dl^{-1}$) and alkaline phosphatase and transaminases ⩽ 2 times the upper normal limit if liver metastases were absent or ⩽ 5 times the upper normal limit if liver metastases were present.

Topotecan was administered orally and intravenously at a dose of 1.5 mg $m^{-2}$. Topotecan as supplied for intravenous infusion was diluted for oral dosage. Each 5 mg vial of topotecan was reconstituted with 10 ml of 5% dextrose injection solution to give a 0.5 mg $ml^{-1}$ solution of the drug. The dose was placed in a beaker containing 100 ml of 5% dextrose injection solution. On day 1, patients were asked to drink the entire contents. Subsequently, an additional 100 ml of 5% dextrose injection solution was added to the beaker and taken by the patient. On day 2 topotecan was administered at the same dose as a continuous 30 min intravenous infusion. The 5 mg vials of topotecan were reconstituted in 5% dextrose and the appropriate volume was then added to a volume of 50 ml of 5% dextrose. The drug was given at approximately the same time of day, between 08.00 and 11.00 h. On both days of drug administration the patients were fasted for 4 h before the time schedule for dosing. Fasting continued for a further 2 h after drug administration on both days. Fluid intake was not restricted.

### *Blood sample collection*

Serial heparinised blood samples (2.8 ml) were collected on both days of drug administration through an indwelling intravenous cannula that was inserted in the arm contralateral to that which was used for the infusion. Blood samples were taken at 0, 10, 20, 30 and 45 min and 1, 1.5, 2.5, 3.5, 4.5, 6.5 and 8.5 h after start of drug administration. After collection, the blood samples were centrifuged immediately for 5 min at 3500 r.p.m. One millilitre of the plasma was immediately transferred to a polypropylene tube containing 4 ml of cold methanol (−20°C) and mixed on a whirl mixer for 15 s. Subsequently, the mixture was centrifuged for 5 min at 3500 r.p.m. and the clear supernatant was transferred to a clean polypropylene tube, closed tightly and stored immediately at −80°C until analysis. Analysis took place within 1 month.

### *Chemicals*

All chemicals were obtained from Baker (Deventer, The Netherlands) and were of analytical grade or higher.

### *Instruments*

The high-performance liquid chromatography (HPLC) apparatus consisted of a pumping device model 6000A (Waters, Milford, MA, USA), a model SP 8880 automated sample injection device (Spectra Physics, Santa Clara, CA, USA), a Perkin Elmer LS40 fluorescence detector (excitation wavelength 381 nm, emission wavelength 527 nm; Perkin Elmer, Norwalk, CT, USA) and a model SP-4290 data analysis system (Spectra Physics). Separation was achieved with a LiChrosorb RP-18 (particle size 5 $\mu$m) column (125 × 4 mm i.d.; Merck, Darmstadt, Germany).

### *Assay of topotecan and hydroxy acid*

Topotecan and the hydroxy acid were analysed simultaneously with an automated reversed-phase HPLC system and fluorescence detection as described by Beijnen *et al.* (1990), with modifications to reduce the lower limit of quantitation (LLQ) (Loos *et al.*, 1996). The LLQ was 0.1 ng $ml^{-1}$ for topotecan as well as for the hydroxy acid. Data were accepted if the deviation from the nominal value of the individual calibration samples and quality controls was <15% (20% at the LLQ).

### *Pharmacokinetic analysis*

The areas under the plasma concentration–time curves (AUC) of topotecan and hydroxy acid were calculated with compartmental and non-compartmental analysis using the Siphar software package release 4.0 (Siphar SIMED, Creteil, France). The trapezoidal method was used for the non-compartmental analysis. The AUC($t$) was calculated up to the latest measured time point. The total AUC (AUC∞) was calculated after extrapolation of the curve to infinity where appropriate using the terminal elimination rate constant $k$. The apparent bioavailability was calculated as the ratio of the AUC($t$) after oral and i.v. administration. All concentration–time profiles were fitted using the extended least squares method (LSM) (Sheiner and Beal, 1985). The volume of distribution at steady state ($V_{d,ss}$) and total plasma clearance (Cl = dose/AUC) were calculated after i.v. administration of topotecan using the AUC extrapolated to infinity (AUC∞). The terminal half-life ($t_{1/2}$) was calculated after oral and i.v. administration as $\ln 2/k$.

Visual inspection of the concentration–time curves, the objective function and the Akaike information criterion (Yamaoka *et al.*, 1978) were applied to choose the optimal model for quantitation of the AUC.

### *Statistical analysis*

The Pearson correlation coefficient was calculated between the ratio of the AUC($t$) of the hydroxy acid and topotecan after oral and i.v. administration and between the ratio of the AUC($t$) of the hydroxy acid after oral and i.v. administration and the bioavailability. Two-sided paired and unpaired $t$-tests were applied to determine any significant difference between the half-lives of topotecan and hydroxy acid after oral and i.v. administration. The influence of the presence of liver metastases and concomitant medication on bioavailability and clearance were evaluated with the Mann–Whitney $U$-test. The relationship between age and bioavailability and clearance was evaluated with the Pearson correlation coefficient and the Wilcoxon rank sum test.

### Results

### *Patients and treatment*

Twelve patients were included in the study (seven males and five females). The mean age was 62 ± 7.5 years (range 46–70). The median performance score was 1 (range 0–2). Nine patients had metastatic colon cancer, two had small-cell lung cancer and one ovarian cancer. Nine patients had documented liver metastases. Five patients did not use concomitant medication, two used a benzodiazepine, one used slow-release morphine and diclofenac, one disopyramide, one alizapride and diclofenac, one atenolol and one atenolol, furosemide, nifedipine, naproxen, budesonide by inhalation, acetylsalicylic acid and nitroglycerine. All patients had been entered in phase II studies applying topotecan in a daily × 5 schedule with cycles repeated every 3 weeks. Oral administration of topotecan was without exception on the first day of one of the 5 day treatment cycles. Neither i.v. nor oral administration was associated with any significant acute side-effect. The oral drinking solution was well tolerated.

### *Pharmacokinetic analysis*

Representative plasma concentration–time curves of topotecan and hydroxy acid after oral and i.v. administration are given in Figure 1. Concentrations of topotecan lactone and hydroxy acid were always undetectable on day 2, before administration of the second dose. In only 6 out of 12 patients visual inspection of the obtained concentration–time profiles of topotecan after oral administration revealed a good fit of the measured plasma concentration–time points, applying extended LSM and one-, two- or three-compartment models [the corresponding correlation coefficients ($r$) were >0.97]. All i.v. profiles of topotecan could be fitted well ($r$>0.97) applying LSM and the outlined models. In ten patients the model using a two-exponential decline of the concentration–time curve resulted in the highest correlation coefficient and lowest Akaike value. In one patient the model with a three-exponential decline of the curve and in one other patient a one-exponential decline resulted in the best fit. In most patients the curves of the hydroxy acid could not be fitted properly using extended LSM, assuming linear pharmacokinetics. The plasma concentration–time curves of the hydroxy acid were higher than those of topotecan in all patients. The pharmacokinetic data are summarised in Tables I and II.

After oral administration the per cent of the AUC extrapolated in eight patients was >20%, therefore the bioavailability was calculated using the ratio of AUC($t$) instead of the AUC extrapolated to infinity (AUC∞). The mean ± s.d. of the AUC($t$) of topotecan after oral administration was 15.18 ± 5.51 ng $h^{-1}$ $ml^{-1}$ (range 9.38–25.37 ng $h^{-1}$ $ml^{-1}$; Table I). The AUC($t$) of topotecan after i.v. administration was 49.87 ± 10.87 ng $h^{-1}$ $ml^{-1}$ (range 32.50–73.68 ng $h^{-1}$ $ml^{-1}$). The AUC∞ of topotecan after i..v. administration was 61.02 ± 10.57 ng $h^{-1}$ $ml^{-1}$ (range 43.70–81.08 ng $h^{-1}$ $ml^{-1}$). The bioavailability was 30% ± 7.7% (range 21%–45%). The coefficient of variation (CV) was 25.4%. If the curves had been extrapolated to infinity, then the bioavailability would have been calculated as 32% ± 11.5%.

The median of the $T_{max}$ was 0.78 h and the range 0.33–2.5 h. The half-life for the initial decline of the plasma concentration–time curve ($t_{1/2\ \infty}$) after i.v. administration was 0.186 ± 0.054 h based on the data for the ten patients that were fitted with a biexponential model.

**Figure 1** Plasma concentration–time curve of topotecan and hydroxy acid in a patient after oral and i.v. administration of 3.2 mg of topotecan. ▲, i.v. topotecan; ●, i.v. hydroxy acid; △, oral topotecan; ○, oral hydroxy acid; closed lines represent topotecan and the dotted lines the hydroxy acid.

The ratio of the AUC($t$) of topotecan and the hydroxy acid after oral administration was $0.63 \pm 0.25$ ($n = 9$) and after i.v. administration $0.72 \pm 0.16$. The correlation coefficient was 0.87 ($P = 0.002$). The ratio of the AUC($t$) of the hydroxy acid after oral and i.v. administration was $0.38 \pm 0.12$ ($n = 9$), which is of the same magnitude as the bioavailability of topotecan. The correlation coefficient between this ratio and the bioavailability was 0.84 ($P = 0.004$).

There was no significant relationship between age or gender and bioavailability. In addition, there was no significant relationship between the presence of liver metastases and the magnitude of the bioavailability.

The $C_{max}$ of the hydroxy acid after oral administration was $7.50 \pm 2.57$ ng ml$^{-1}$ (range $4.66 \pm 12.32$ ng ml$^{-1}$) and after i.v. administration $18.97 \pm 2.44$ ng ml$^{-1}$ (range 15.35–24.12 ng ml$^{-1}$). The terminal $t_{1/2}$ after oral administration was $2.82 \pm 0.85$ h and after i.v. administration $3.22 \pm 0.73$ h (not significantly different).

### Discussion

The present study is the first to provide data on the systemic exposure of topotecan after oral administration. Chronic administration of topotecan resulted in an enhanced antineoplastic activity (Giovanella *et al.*, 1989; Burris *et al.*, 1992; Houghton *et al.*, 1992; Rowinsky *et al.*, 1992; Hochster *et al.*, 1994; Verweij *et al.*, 1993). The concept of chronic administration is to some extent applied in a large number of phase II studies that are currently in progress using a daily ×5 intravenous infusion of 30 min or 21 day continuous infusion. Topotecan may show an even more pronounced anti-tumour response if the exposure duration is even further prolonged (Hochster *et al.*, 1994). It would increase the convenience for the patient substantially if the drug on a chronic treatment schedule could be taken orally.

The apparent bioavailability was determined in 12 patients with various types of solid tumours. The bioavailability ranged from 21% to 45%. The term apparent bioavailability has been used because topotecan undergoes a reversible, pH-dependent conversion to the hydroxy acid at physiological pH and the standard equation for the calculation of bioavailability no longer applies. The correct equations contain terms for the AUC of topotecan, and hydroxy acid after i.v. administration of the hydroxy acid itself. The standard equation for bioavailability, as used in this study, is a function not only of dose and input rate, but also of conversion clearance. The accuracy of the apparent bioavailability data will depend, to a large extent, on the magnitude of the conversion clearance, for which no *in vivo* data on topotecan are available. Even although at physiological pH the formation of the hydroxy acid is the predominant reaction, experiments on camptothecin in the rat have shown that the lactone can be formed following the i.v. administration of the hydroxy acid (Scott *et al.*, 1994). As camptothecin and topotecan have the same basic ring structure, it is highly likely that topotecan could also be formed following administration of the topotecan hydroxy acid. The bioavailability was determined after oral administration on day 1 and i.v. administration on day 2. This strategy was followed to deviate as little as possible from the phase II daily ×5 schedule that has documented therapeutic activity in several tumour types. The approach is justified

**Table II** Mean pharmacokinetic data of topotecan lactone

| | *Oral* | | *Intravenous* | |
|---|---|---|---|---|
| Cl (ml min$^{-1}$) | – | | 824±117 (535–1068) | ($n$=12) |
| $V_{d,ss}$ (l) | – | | 128±37.1 (86–231) | ($n$=12) |
| $t_{1/2\beta}$ (h) | 2.43±1.15 (1.02–3.20) | ($n$=10) | 2.40±0.38 (1.72–2.93) | ($n$=12) |

Mean ± s.d. and range are given. Cl, total plasma clearance. $V_{d,ss}$, volume of distribution at steady state.

**Table I** Individual pharmacokinetic data of topotecan lactone and hydroxy acid

| *Patient (no.)* | *Dose (mg)* | $T_{max}$, *O (h)* | *Topotecan* $C_{max}$, *O (ng ml$^{-1}$)* | $C_{max}$, *i.v. (ng ml$^{-1}$)* | *AUC(t), O (ng h$^{-1}$ ml$^{-1}$)* | *AUC(t), i.v. (ng h$^{-1}$ ml$^{-1}$)* | *AUC∞ i.v. (ng h$^{-1}$ ml$^{-1}$)* | F (%) | *Hydroxy acid AUC(t), O (ng h$^{-1}$ ml$^{-1}$)* | *AUC(t), i.v. (ng h$^{-1}$ ml$^{-1}$)* |
|---|---|---|---|---|---|---|---|---|---|---|
| 1 | 2.7 | 0.5 | 5.95 | 41.08 | 10.48 | 49.17 | 64.49 | 21 | NE | 59.86 |
| 2 | 2.8 | 1.03 | 5.93 | 35.92 | 9.38 | 32.50 | 43.70 | 30 | 12.20 | 37.08 |
| 3 | 3.2 | 0.33 | 5.16 | 43.86 | 10.85 | 42.37 | 56.34 | 25 | 15.10 | 60.72 |
| 4 | 3.0 | 1.13 | 5.45 | 41.81 | 18.81 | 49.37 | 58.15 | 38 | 47.96 | 90.79 |
| 5 | 2.7 | 0.52 | 5.71 | 33.40 | 11.25 | 37.96 | 46.64 | 30 | NE | 56.34 |
| 6 | 3.0 | 0.55 | 7.14 | 47.18 | 12.75 | 55.09 | 69.62 | 23 | 29.30 | 100.83 |
| 7 | 2.8 | 1.03 | 4.71 | 40.35 | 9.79 | 45.31 | 59.53 | 22 | NE | 65.07 |
| 8 | 2.8 | 2.5 | 5.08 | 41.41 | 21.98 | 48.57 | 52.04 | 45 | 63.99 | 103.25 |
| 9 | 3.1 | 0.5 | 6.48 | 41.21 | 12.46 | 55.00 | 66.00 | 23 | 19.22 | 57.70 |
| 10 | 2.6 | 1.5 | 6.67 | 42.11 | 25.37 | 73.68 | 81.08 | 34 | 51.47 | 121.14 |
| 11 | 2.8 | 1.0 | 5.38 | 25.35 | 21.56 | 62.31 | 70.14 | 35 | 19.01 | 63.77 |
| 12 | 2.7 | 0.5 | 6.93 | 19.57 | 17.00 | 46.61 | 64.55 | 36 | 24.46 | 62.48 |
| Mean | | | 5.88 | 37.77 | 15.18 | 49.87 | 61.02 | 30 | 31.41 | 73.25 |
| s.d. | | | 0.78 | 8.04 | 5.51 | 10.87 | 10.57 | 7.7 | 18.47 | 24.69 |

O, oral; i.v., intravenous; dose, absolute dose (oral = i.v.); $T_{max}$, time to maximal plasma concentration; $C_{max}$, maximal plasma concentration; AUC($t$), area under the curve up to the latest measured time point; AUC∞, area under the curve extrapolated to infinity; NE, not evaluated.

because there is no carryover of topotecan into the second treatment period. In addition, topotecan is not metabolised and therefore cannot induce its own elimination. Plasma samples were collected up to 8 h after oral administration. This was slightly too short to extrapolate the AUC up to infinity. The sampling time was determined based on preclinical data on the oral administration in dogs and on the elimination pharmacokinetics after i.v. administration in man. The bioavailability after oral administration in dogs was 35.7% ± 16.3% (unpublished data), which is close to the result obtained in patients. The calculation of the bioavailability using the AUC∞ resulted in only a marginally higher value of 32% instead of 30%. Apparently, the applied ratio of AUC($t$) gives a good estimate of the bioavailability.

The $t_{1/2}\ \beta$ in the patient with the highest bioavailability of 45% was 1.8 h after i.v. and 3.2 h after oral administration. This difference was relatively large in comparison with the data obtained in the other patients. This patient had a large tumour in the upper part of the abdomen. It cannot be excluded that this extensive tumour mass has influenced the rate of passage of topotecan through the gastrointestinal tract and thereby the magnitude of the absorption. The $T_{max}$ of this patient was 2.5 h, which is delayed compared with the range of the other 11 patients (0.33 – 1.5 h).

The CV of the AUC($t$) after oral administration was 36.6% and after i.v. administration 21.8%. Hence, oral administration increased the interpatient difference in systemic exposure markedly. It has to be elucidated in follow-up studies whether this variation has clinical implications. In addition, the intra-patient variability needs to be determined in future studies.

The CV of the plasma clearance of topotecan after i.v. administration was 18.8%. The range of the data was of the same order as reported by Rowinsky *et al.* (1992) and Verweij *et al.* (1993).

No relationships were found between patient characteristics such as age, gender, performance score and the bioavailability. In addition, the presence of liver metastases or concomitant drugs did not seem to be related to the magnitude of the bioavailability.

The ratio of topotecan and hydroxy acid was of the same order after oral and i.v. administration. There would appear only two reasonable explanations for the observed data: either topotecan is poorly absorbed from the gastrointestinal tract or topotecan is well absorbed but a large part of the dose is converted presystemically in the gut into the hydroxy acid, which is itself not absorbed to any appreciable extent. The good water solubility of topotecan coupled with the rapid absorption would suggest that the second explanation can best describe the data obtained in this study. There is no evidence for the formation of other metabolites that may explain the observed data.

The bioavailability of topotecan after oral administration illustrates significant systemic exposure to the drug which may enable chronic oral treatment.

### References

BEIJNEN JH, SMITH BR, KEIJER WJ, VAN GIJN R, TEN BOKKEL HUININK WW, VLASVELD LT, RODENHUIS S AND UNDERBERG WJ. (1990). High performance liquid chromatographic analysis of the new antitumour drug SK&F 104864 (NSC-609699) in plasma. *J. Pharm. Biomed. Anal.*, **8**, 789 – 794.

BLANEY SM, BALIS FM, COLE DE, CRAIG C, REID JM, AMES MM, KRAILO M, REAMAN G, HAMMOND D AND POPLACK DG. (1993). Pediatric phase I trial and pharmacokinetic study of topotecan administered as a 24-hour continuous infusion. *Cancer Res.*, **53**, 1032 – 1036.

BURRIS HA, HANAUSKE AR, JOHNSON RK, MARSHALL MH, KUHN JG, HILSENBECK SG AND VON HOFF DD. (1992). Activity of topotecan, a new topoisomerase I inhibitor, against human tumour colony-forming units *in vitro*. *J. Natl Cancer Inst.*, **84,** 1816 – 1820.

CREEMERS GJ, LUND B AND VERWEIJ J. (1994). Topoisomerase I inhibitors: topotecan and irinotecan. *Cancer Treatm. Rev.*, **20,** 73 – 96.

GIOVANELLA BC, STEHLIN JS, WALL ME, WANI MC, NICHOLAS AW, LIU LF, SILBER R AND POTMESIL M. (1989). DNA topoisomerase I-targeted chemotherapy of human colon cancer in xenografts. *Science*, **246,** 1046 – 1048.

HOCHSTER H, LIEBES L, SPEYER J, SORICH J, TAUBES B, ORATZ R, WERNZ J, CHACHOUA A, RAPHAEL B, VINCI RZ AND BLUM RH. (1994). Phase I trial of low-dose continuous topotecan infusion in patients with cancer: an active and well-tolerated regimen. *J. Clin. Oncol.*, **12,** 553 – 559.

HOUGHTON PJ, CHESHIRE PJ, MYERS L AND HOUGHTON JA. (1992). Evaluation of 9-dimethylaminomethyl-10-hydroxycamptothecin (topotecan) against xenografts derived from adult and childhood solid tumours. *Cancer Chemother. Pharmacol.*, **31,** 229 – 239.

HSIANG YH AND LIU LF. (1988). Identification of mammalian DNA topoisomerase I as an intracellular target of the anticancer drug camptothecin. *Cancer Res.*, **48,** 1722 – 1726.

JOHNSON RK, MCCABE FL, GALLAGHER G, WOOD J, GALEF J AND HERZBERG RP. (1992). Comparative efficacy of topotecan, irinotecan and of 9-aminocamptothecin in preclinical models. *Ann. Oncol.*, 3 (suppl. 1),105.

KINGSBURY WD, BOEHM JC, JAKAS DR, HOLDEN KG, HECHT SM, GALLAGHER G, CARANFA MJ, MCCABE FL, FAUCETTE LF, JOHNSON RK AND HERTZBERG RP. (1991). Synthesis of watersoluble (aminoalkyl) camptothecin analogues: Inhibition of topoisomerase I and antitumour activity. *J. Med. Chem*, **34,** 98 – 107.

LOOS WJ, STOTER G, VERWEIJ J AND SCHELLENS JHM. (1996). Sensitive high-performance liquid chromatographic fluorescence assay for the quantitation of topotecan (SKF104864-A) and lactone ring-opened product (hydroxy-acid) in human plasma and urine. *J. Chromatogr. Biomed. Appl.* (in press).

MILLER AA, HARGIS JB, FIELDS S, LILENBAUM RC, ROSNER GL AND SCHILSKY RL. (1993). Phase I study of topotecan and cisplatin in patients with advanced cancer (CALGB 9261). *Proc. Am. Soc. Clin. Oncol.*, **12,** 399.

ROTHENBERG ML, BURRIS HA, ECKARDT JR, RINALDI DA, WEISS GR, SMITH S, JONES K, JOHNSON RK AND VON HOFF DD. (1993). Phase I/II study of topotecan plus cisplatin in patients with non-small cell lung cancer (NSCLC). *Proc. Am. Soc. Clin. Oncol.*, **12,** 156.

ROWINSKY EK, GROCHOW LB, HENDRICKS CB, ETTINGER DS, FORASTIERE AA, HUROWITHZ LA, MCGUIRE WP, SARTORIUS SE, LUBEJKO BGM KAUFMANN SH AND DONEHOWER RD. (1992). Phase I and pharmacologic study of topotecan: A novel topoisomerase I inhibitor. *J. Clin. Oncol.*, **10,** 647 – 656.

SCOTT DO, BINDRA DS AND STELLA VJ. (1994). Pharmacokinetics of the lactone and carboxylate forms of 20(S)-camptothecin in anaesthetized rats. *Pharm. Res.*, **10,** 1451 – 1457.

SHEINER LB AND BEAL SL. (1985). Pharmacokinetic parameter estimates from several least squares procedures: superiority of extended least squares. *J. Pharmacokinet. Biopharm.*, **13,** 185 – 201.

VERWEIJ J, LUND B, BEIJNEN JH, PLANTING ASTh, DE BOER-DENNERT M, KOIER I, ROSING H AND HANSEN H. (1993). Phase I and pharmacokinetics study of topotecan, a new topoisomerase I inhibitor. *Ann. Oncol.*, **4,** 673 – 678.

WALL JG, BURRIS H, VON HOFF D, RODRIGUEZ G, KNEUPER-HALL R, SHAFFER D, O'ROURKE T, BROWN T, WEISS G, CLARK G, MCVEA S, BROWN J, JOHNSON RK, FRIEDMAN C, SMITH B, MANN WS AND KUHN J. (1992). A phase I clinical and pharmacokinetic study of the topoisomerase I inhibitor topotecan (SK&F 104864) given as an intravenous bolus every 21 days. *Anticancer Drugs*, **3,** 337 – 342.

YAMAOKA K, NAKAGAWA T AND UNO T. (1978). Application of Akaike's information criterion (AIC) in the evaluation of linear pharmacokinetic equations. *J. Pharmacokinet. Biopharm.*, **6,** 165 – 175.